# Comparative Genomics of Two Sequential *Candida glabrata* Clinical Isolates

**DOI:** 10.1534/g3.117.042887

**Published:** 2017-06-28

**Authors:** Luis Vale-Silva, Emmanuel Beaudoing, Van Du T. Tran, Dominique Sanglard

**Affiliations:** * Institute of Microbiology, University of Lausanne, CH-1011, Switzerland; † Lausanne University Hospital, CH-1011, Switzerland; ‡ Center for Integrative Genomics, Lausanne Genomic Technologies Facility, CH-1015, Switzerland; § Vital-IT Group, SIB Swiss Institute of Bioinformatics, CH-1015 Lausanne, Switzerland

**Keywords:** fungal pathogens, genome comparisons, drug resistance, adhesins, Genome Report

## Abstract

*Candida glabrata* is an important fungal pathogen which develops rapid antifungal resistance in treated patients. It is known that azole treatments lead to antifungal resistance in this fungal species and that multidrug efflux transporters are involved in this process. Specific mutations in the transcriptional regulator *PDR1* result in upregulation of the transporters. In addition, we showed that the *PDR1* mutations can contribute to enhance virulence in animal models. In this study, we were interested to compare genomes of two specific *C. glabrata*-related isolates, one of which was azole susceptible (DSY562) while the other was azole resistant (DSY565). DSY565 contained a *PDR1* mutation (L280F) and was isolated after a time-lapse of 50 d of azole therapy. We expected that genome comparisons between both isolates could reveal additional mutations reflecting host adaptation or even additional resistance mechanisms. The PacBio technology used here yielded 14 major contigs (sizes 0.18–1.6 Mb) and mitochondrial genomes from both DSY562 and DSY565 isolates that were highly similar to each other. Comparisons of the clinical genomes with the published CBS138 genome indicated important genome rearrangements, but not between the clinical strains. Among the unique features, several retrotransposons were identified in the genomes of the investigated clinical isolates. DSY562 and DSY565 each contained a large set of adhesin-like genes (101 and 107, respectively), which exceed by far the number of reported adhesins (63) in the CBS138 genome. Comparison between DSY562 and DSY565 yielded 17 nonsynonymous SNPs (among which the was the expected *PDR1* mutation) as well as small size indels in coding regions (11) but mainly in adhesin-like genes. The genomes contained a DNA mismatch repair allele of *MSH2* known to be involved in the so-called hyper-mutator phenotype of this yeast species and the number of accumulated mutations between both clinical isolates is consistent with the presence of a *MSH2* defect. In conclusion, this study is the first to compare genomes of *C. glabrata* sequential clinical isolates using the PacBio technology as an approach. The genomes of these isolates taken in the same patient at two different time points exhibited limited variations, even if submitted to the host pressure.

Infectious diseases caused by fungal pathogens represent a major threat to human health. It is estimated that ∼2 billion people suffer from several forms of fungal diseases worldwide, from which ∼1.5 million deaths will occur every year (Brown *et al.* 2012). Invasive fungal infections cause on average 50% mortality, with total number of deaths comparable to tuberculosis and malaria (Brown *et al.* 2012). The most problematic fungal pathogens include *Candida*, *Aspergillus*, and *Cryptococcus* species (Richardson and Lass-Flörl 2008). Within *Candida* species, *Candida albicans* and *C. glabrata* are known as the most-recovered species from infected patients (Marchetti *et al.* 2004). The current reports on the epidemiology of fungal diseases and their associated impact on human health reflect that the available therapeutic options, which include antifungal drugs, have limited efficacy. Only four major classes of antifungal drug are used to treat patients including azoles, polyenes, echinocandins, and pyrimidine analogs. Each of these agents have specific cellular targets and are used in several formulations with different derivatives (Ruhnke 2014). In general, most *Candida* species are susceptible to these drugs *in vitro*.

However, the use of antifungal agents has resulted in the inevitable occurrence of antifungal resistance. Antifungal resistance in a specific fungal isolate can be defined as a lack of activity of a given drug at concentrations that are higher than in a wild-type isolate. Antifungal resistance has been reported for virtually all existing antifungal agents and major fungal pathogens (Perlin *et al.* 2015). Frequencies at which antifungal resistance occur in hospitalized patients are variable based on data collected by population-based surveillance programs and available for major fungal pathogens including *C. albicans* and *C. glabrata*. In general, antifungal resistance rates for *C. albicans* are low. In a study performed between 2008 and 2011, resistance to fluconazole (cut-off: ≥64 µg/ml) or echinocandins (cut-off: ≥4 µg/ml) ranged from between 1 and 2% in bloodstream isolates (Cleveland *et al.* 2012). Resistance rates in *C. glabrata* are much higher: *C. glabrata* infections as a cause of invasive candidiasis increased from 18% of all BSI isolates in 1992–2001 to 25% in 2001–2007. Fluconazole resistance rates in *C. glabrata* increased from 9 to 14% in the same time period (cut-off: ≥64 µg/ml) (Pfaller *et al.* 2003, 2009). Resistance of *C. glabrata* to the class of echinocandins is also increasing: within a 10-yr period (2001–2010) in a US hospital, the echinocandin resistance rate increased from 4.9 to 12.3% (Alexander *et al.* 2013). Similar trends are documented in Europe, however resistance rates are reported to be between 1 and 4% (Arendrup and Perlin 2014).

Recent studies have also documented the occurrence of multidrug resistance not only in *C. albicans* but also in *C. glabrata* (Sanglard 2016). *C. glabrata* cross-resistance between azoles and candins has captured more attention recently (Perlin 2015). For example, among echinocandin-resistant isolates sampled between 2008 and 2013 in two US surveillance hospital sites, 36% were also resistant to azoles (Pham *et al.* 2014). Between 2005 and 2013 in another US site, 10.3% *C. glabrata* isolates from cancer patients were resistant to caspofungin, from which ∼60% were cross-resistant to azoles (Farmakiotis *et al.* 2014). Intriguingly, the later study reported that caspofungin exposure alone could induce multidrug resistance without azole exposure. While *C. glabrata* azole resistance is mediated principally by efflux-dependent mechanisms, candin resistance is the consequence of mutations in the genes (*FKS1* and *FKS2*) encoding the candin target enzyme (β-glucan synthase) (Perlin 2015; Vale-Silva and Sanglard 2015). It has been shown recently that *C. glabrata* can exhibit high mutation rates in the presence of antifungal agents. This property is based on the decreased activity of the DNA repair machinery that exists in specific clinical isolates (Healey *et al.* 2016b).

We have shown in the past years that *C. glabrata* azole resistance is coupled with selective fitness advantages in animal models of infection (Ferrari *et al.* 2009; Vale-Silva *et al.* 2013). Specific isolates exhibit upregulation of the adhesin *EPA1* when the transcriptional activator responsible for azole resistance *CgPDR1* is mutated. When *EPA1* is upregulated, the consequence is that *C. glabrata* exhibits increased adherence to host cells, which is one of the factors that contributes to enhance virulence of this yeast species which develops azole resistance (Vale-Silva *et al.* 2016). Our recent data also highlighted that *C. glabrata* can exhibit different degrees of adhesion to host cells. Given that adhesins are implicated in this process, it becomes relevant to address the repertoire of adhesins in specific *C. glabrata* lineages. Genome data are critical to achieve this goal, however the only available *C. glabrata* genome is up to now from the isolate CBS138 (Dujon *et al.* 2004). It is estimated that *C. glabrata* contains 63 ORFs with adhesin properties (de Groot *et al.* 2013). In a recent study, we compared CBS138 to other clinical isolates and found that a specific isolate (DSY562) exhibited the much higher adherence capacities to epithelial cells as compared with CBS138. This result was not dependent on *EPA1* expression since DSY562 expressed *EPA1* at much lower levels than CBS138.

These results stimulated our interest into the acquisition of the DSY562 genome. At the same time, it became also pertinent to compare the genome of this isolate with one (DSY565) that acquired azole resistance within a time-lapse of 50 d from DSY562 in a patient with oropharyngeal candidiasis (Sanglard *et al.* 1999). In this way, evolutionary pressure on the derived isolate could be estimated at the level of genome differences.

Here we report genome sequences from both related isolates. We used several approaches to resolve the genome with high accuracy. We analyzed the occurrence of polymorphisms between the two strains and also addressed the repertoire of genes encoding adhesins.

## Materials and Methods

### Strains and media

Strains DSY562 and DSY565 were reported earlier (Sanglard *et al.* 1999) and were grown in YEPD at 30° under constant agitation.

### DNA isolation

Genomic DNA from DSY5652 and DSY565 was obtained by the spheroplasting method as previously described (Calabrese *et al.* 2000).

### Multilocus sequence typing

Multilocus sequence typing (MLST) was performed according to the study of Dodgson *et al.* (2003). Sequencing data of individual loci were analyzed using an online web tool (http://cglabrata.mlst.net) to determine the sequence type (ST).

### Genome sequencing

To produce high-quality genomic DNA from *C. glabrata* isolates, overnight cultures (5 ml) were first grown in YNB minimal medium [0.67% yeast nitrogen base (Dicfo) with 2% glucose] at 30° under constant shaking to obtain 2 × 10^8^ cells for each strain. The DNA isolation protocol followed instructions of the Gentra Puregene Yeast/Bact kit (Qiagen) with slight modifications. First, yeast cell lysis was performed with Zymolyase 100T (3 µg/µl end concentration) for 30 min at 37°. In addition, after RNAse A treatment of precipitated nucleic acids, phenol/chloroform extractions were carried out according to recommendations issued by Pacific Bioscience (PacBio SampleNet—Shared Protocol) for the use of phenol/chloroform/isoamyl alcohol. After final precipitation with NH_4_OAc and several washes with 80% alcohol, the genomic DNA was dissolved carefully in a small volume of elution buffer (10 mM Tris-HCl, pH 8.5). Aliquots of 5 μg of extracted, high-quality genomic DNA was diluted to 150 μl using elution buffer at 30 μg/μl. Long insert SMRTbell template libraries were prepared (20-kbp insert size) according to PacBio protocols. In total, nine SMRT cells per strain were sequenced using P4 and P5 polymerase binding and C2 and C3 sequencing kits with 120 min (five first SMRT cells) and 180 min acquisition on PacBio RSII. *De novo* genome assemblies were produced using PacBio’s SMRT Portal (v2.0.0) and the hierarchical genome assembly process (HGAP version 3.0), with default settings and a seed read cut-off length of 6000 bp.

Illumina-based genome sequencing was performed by FASTERIS SA (Plan-les-Ouates, Switzerland). Briefly, 4 µg of each DSY562 and DSY565 genomic DNA was fragmented by nebulization and the genomic libraries were prepared following the procedure described in the Genomic DNA Sample Prep Kit (Illumina, FC-102-1003). To obtain inserts of 300 bp, a size selection was performed on agarose gel before a 10-cycle PCR amplification. The resulting libraries were sequenced in a 2 × 38-bp run on a Genome Analyzer IIx using the Sequencing Kit v4 (Illumina).

### Genome comparisons

The final PacBio assemblies of DSY562 and DSY565 were analyzed using Geneious (9.1.8). Genome comparisons were performed on the basis of each chromosome’s homolog using the Mauve aligner plug in (progressive Mauve algorithm) (Darling *et al.* 2004). The alignments were next inspected with a SNP discovery tool integrated in the software.

For comparisons between CBS138 and the Illumina-based genome sequencing of DSY562 and DSY565, reads were imported in the CLC genomic workbench software (v.9.5.2) (Qiagen). Variant detection was performed from the mapped reads using both probabilistic and quality-based variant detection tools available in CLC. A pipeline of the analysis of genome data are included in Supplemental Material, Figure S4.

### RNAseq

RNA extractions from the *C. glabrata* strains DSY562 and DSY565 were carried out as previously published (Ferrari *et al.* 2011). The strains were grown to logarithmic phase in 5 ml YEPD medium [1% Bacto peptone (Difco), 0.5% yeast extract (Difco), 2% glucose (Fluka)] at 30° under constant shaking. RNA libraries for RNAseq were prepared with a TruSeq Stranded Total RNA Library Prep Kit (Illumina). The resulting libraries were processed using the Illumina TruSeq PE cluster kit v3 reagents and sequenced on the Illumina HiSequation 2500 system using TruSeq SBS Kit v3 reagents. Sequencing data were processed using Illumina Pipeline software version 1.82 to obtain fastq files.

### Sanger sequencing

Sequencing reactions were carried with a BigDye Terminator DNA Sequencing Kit (Thermo Fisher Scientific, Waltham, MA) and analyzed with a 3130XL genetic analyzer (Applied Biosystems). Primers used are listed in Table S1. Forward and reverse tag primer tails (5′-CGACGCCCGCTGATA-3′ and 5′-GTCCGGGAGCCATG-3′) were added to each primer pair (labeled by suffixes “-R” and “-F”). PCR were performed on DSY562 and DSY565 DNA with the forward and reverse primers which was followed by sequencing with the tag primers.

### Adhesins predictions

DSY562 ORFs were analyzed with the help of the software FungalRV (http://fungalrv.igib.res.in/index.html), which helps to predict the occurrence of adhesin-like proteins in fungal genomes. To select for potential adhesins, a value of 0.5 was set as a threshold, as recommended (Chaudhuri *et al.* 2011). The list of potential adhesins was further subjected to several additional tests including a search for PFAM domains (http://pfam.xfam.org/search#tabview=tab1) and the presence of specific signatures relevant to the detection of adhesin-like proteins. These signature included (i) the presence of signal peptides and absence of internal transmembrane domains using the online programs SignalP and TMHMM (http://www.cbs.dtu.dk/services/); (ii) the presence of consensus sequence for GPI anchor ([NSGDAC] – [GASVIETKDLF] – [GASV] – X(4,19) – [FILMVAGPSTCYWNQ](10)>), as recommended by Weig *et al.* (2004); (iii) the presence of AWP motifs (VSHITT) (de Groot *et al.* 2013); (iv) the presence of EPA motifs (EPA CC: CX(27)CX(40)CX(44)DDX(13)CC) and EPA: GCSX(8,9)GL) following Diderrich *et al.* (2015) and Gabaldón *et al.* (2013); and (v) the presence of PIR motifs (Q[IV]XDGQ[IVP]Q) following (de Groot *et al.* 2008).

### Protein alignments

Protein alignments were performed with a Web tool available at http://mafft.cbrc.jp/alignment/server (MAFFT version 7) and default settings. A phylogenetic tree was constructed from the alignment produced from the tool. The produced alignments were next visualized after average linkage (UPGMA) and a bootstrapping with 1000 resamplings.

### Detection of splicing junctions

RNAseq data obtained from DSY562 and DSY565 were imported as fastq files into Geneious (9.1.8). Splice junctions were detected using the TopHat RNAseq aligner (Trapnell *et al.* 2009).

### Counterclamped homogeneous electric field (CHEF) analysis

Two or three colonies of each yeast strain were inoculated in YEPD liquid medium and incubated overnight at 30° with agitation. A culture volume corresponding to 10^9^ cells was pelleted at 3000 × *g* for 5 min, and cells were washed in 5 ml 50 mM EDTA (pH 9). The pellet was resuspended in 330 µl 50 mM EDTA. Next, 110 µl of SCE [1 M sorbitol, 10 mM EDTA, 100 mM sodium citrate (pH 5.8), adjusted with citric acid] was added and then completed with 5% β-mercaptoethanol and Zymolyase 100T (100 units/ml) (solution I). The solution was gently mixed with 560 µl of GTG agarose and rapidly distributed into molds of 10 by 5 by 2 mm. The solidified plugs were immersed in solution II [450 mM EDTA, 10 mM Tris-HCl (pH 8), 7.5% β-mercaptoethanol] and incubated overnight at 37° without agitation. Solution II was replaced by solution III [450 mM EDTA, 10 mM Tris-HCl (pH 8), 1% *N*-lauryl- Sarkosyl, and 150 mg/ml proteinase K]. Plugs were incubated for 6 hr at 65° without agitation and kept for 10 min on ice. Finally, plugs were transferred to a 0.5 M EDTA (pH 9) solution and stored at 4° for several months. One-third of the plug was loaded into wells of 0.6% GTG agarose gel in 0.5% Tris-borate-EDTA. The gel was then placed into the electrophoresis chamber of a CHEF DR II (Bio-Rad, Zürich, Switzerland) apparatus. Migration was performed in 0.5% Tris-borate-EDTA at 14° with the following steps: block 1, 60- to 120-min switch, 6 V/cm, and 120° over 24 hr; block 2, 120- to 300-min switch, 4.5 V/cm, and 120° over 22 hr. After gel electrophoresis, gels were stained with ethidium bromide to reveal DNA under ultraviolet light.

### Data availability

Strains described here are available upon request. PacBio assemblies are available under Bioproject PRJNA374542 (SRR5298594–SRR5298602 for DSY565, SRR5298595 and SRR5298512–SRR5298519 for DSY562). RNAseq data are available under Bioproject PRJNA374632 (SRR5296197–SRR5296202). Paired-end reads from Illumina-based genome sequencing are available under Bioproject PRJNA374542 (SRR5459192–SRR5459193). The final assembled genomes for DSY562 and DSY565 and their annotations are available under accession numbers MVOE00000000 and MVOF00000000, respectively.

## Results

### Genome data acquisition

*C. glabrata* DSY562 and DSY565 are paired clinical isolates that were isolated from an HIV-positive patient presenting oropharyngeal candidiasis. DSY565 was isolated after 50 d of fluconazole therapy and exhibited azole resistance (Sanglard *et al.* 1999) (Table 1). The relationship between the two isolates was assessed at this time by RFLP which concluded a high relationship between the isolates. Before undertaking genome sequencing, both isolates were subjected to MLST typing according to Dodgson *et al.* (2005) (Table 1), and thus the high relationship between both isolates was confirmed.

**Table 1:** Characteristics of DSY562 and DSY565

*C. glabrata* Clinical Strains	ST	MIC (µg/ml)			*PDR1* Mutation	Time Elapsed (d)
		Fluconazole	Voriconazole	Amphotericin B		
DSY562	ST8	4	0.25	0.5	—	—
DSY565	ST8	64	1	0.5	L280F	50

ST, sequence type.

Genome sequencing was first approached by Illumina HiSequation 38-bp, paired-end sequencing. Briefly, trimmed reads of both DSY5652 and DSY565 were aligned to the reference genome of CBS138 using the software CLC Genomics Workbench. In both cases, ∼95% of reads aligned to the CBS genomes and coverage was 72- to 64-fold for DSY562 and DSY565 data, respectively. Details on comparisons can be found in File S1 and File S2. Both comparisons yielded a high number of changes (85,711–85,986 including SNPs and indels) and resulted in ∼10,390 nonsynonymous changes as compared to CBS138. Given that the genomes were aligned and compared directly to CBS138, they did not reflect actual genome rearrangements of strains DSY562 and DSY565. We therefore undertook an alternative genome-sequencing approach using PacBio technologies enabling *de novo* assembly of large reads. Since this technology is prone to random intrinsic high error rates, we aimed to reach genome sequencing coverage between 200- and 300-fold, which therefore required the use of several sequencing SMRT cells. The PacBio data are summarized in Table 2. As summarized in this table, the number of major assembled contigs (size >20 kb) were 14 and 15 for DSY562 and DSY565, respectively. N50s were 1.06 and 1.17 Mb for both isolates, thus highlighting the high quality of the assemblies. Table 3 summarizes the sums of nucleotides for each assembled contig, which were assigned to chromosomes according to the current CBS138 genome data (see below). DSY562 and DSY565 accounted for totals of 12,729,188 and 12,728,164 nucleotides. This represents a difference of 1024 nucleotides between the two genomes. The difference in nucleotide counts for major contigs (including mitochondrial DNA) is larger (58,400 nucleotides), however this is compensated for by nucleotide counts of the small contigs which could not be consistently assembled to larger contigs.

**Table 2:** PacBio sequencing analysis of DSY562 and DSY565

	PacBio		
Strain/assembly type	DSY562	DSY565	CBS138
SMRT cells	9 (8 P4C2 + 1 P5C3)	9 (8 P4C2 + 1 P5C3)	—
Number of bases (Gb)	3.4 (coverage: 261×)	2.8 (coverage: 215×)	—
Mean read length (kb)	6.5	5.7	—
Major contigs (including mitochondrial DNA)	14	15	14
Minor contigs (4–20 kb)	5	9	—
Total of assembled bases	12,729,188	12,728,164	12,338,305
Mitochondrial genome	20,086	20,086	20,063
N50	1,178,607	1,064,616	—

**Table 3:** Details of PacBio assembly of DSY562 and DSY565

DSY562	Length (bp)	DSY565	Length (bp)
Chr A	482,027	Chr A	482,027
Chr B	511,306	Chr B	511,306
Chr C	646,952	Chr C	628,526
Chr D-L	695,320	Chr D-L	695,913
Chr E	711,760	Chr E	712,009
Chr F-L-I	1,594,519	Chr F-L-I	1,594,552
Chr G	1,026,668	Chr G	1,009,061
Chr H	1,064,558	Chr H	1,064,559
Chr I-L-D	752,897	Chr I-L-D	744,977
Chr J	1,234,082	Chr J1	1,048,338
		Chr J2	181,774
Chr K	1,305,194	Chr K	1,305,194
Chr L	1,178,610	Chr L	1,176,450
Chr M-J	1,424,660	Chr M-J	1,419,287
Mitochondrial DNA	20,086	Mitochondrial DNA	20,086
Total Chr + mitochondrial DNA	12,648,639		12,594,059
unitig13_20kb	20,067	unitig5_7kb	7847
unitig5_14kb	14,784	unitig6_11kb	11,645
unitig2_20Kb	23,113	unitig12_22kb	22,969
unitig20_10kb	10,815	unitig16_17kb	17,373
unitig16_11kb	11,770	unitig17_9kb	9902
		unitig18_13kb	13,680
		unitig20_14kb	14,791
		unitig22_16kb	16,821
		unitig32_19kb	19,077
Total small contigs	80,549	Total small contigs	134,105
Total all contigs	12,729,188		12,728,164

### Genome comparisons between clinical strains and CBS138

**Figure 1:**
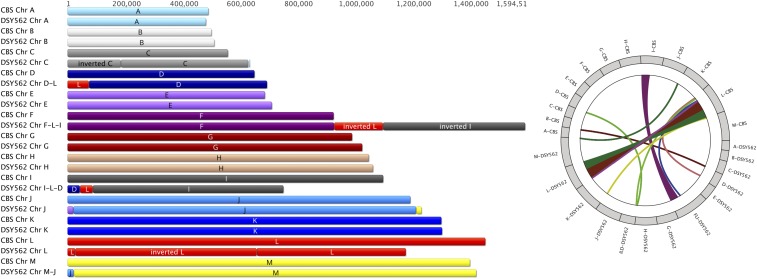
Chromosome structures of DSY562 as compared to CBS138. A circular map representing the major translocations between the CBS138 and DSY562 genomes was produced using J-Circos v2 (An *et al.* 2015).

**Figure 2:**
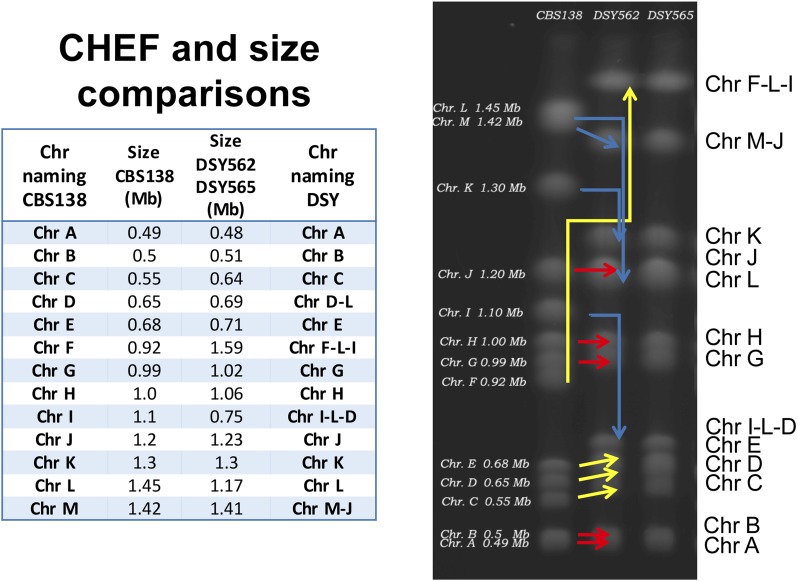
CHEF chromosome separations of isolates CBS138, DSY562, and DSY565. Chromosome sizes are indicated for CBS138 and were extracted from public databases. Colored →’s show the size changes between the CBS and DSY562/DSY565 genomes. Red, yellow, and blue colors signify size conservation, increased sizes, and decreased sizes between CBS138 and the two different genomes, respectively. The attached table lists the chromosome sizes deduced from genome data of CBS138 and DSY562/DSY565. Plotting the relative migration distance (Rf values) of known CBS138 chromosome sizes with the obtained sizes of DSY562 and DSY565 chromosomes yielded an *R*^2^ value of 0.981.

Assembled contigs almost reconstituted the entire set of chromosomes that is known from the CBS138 genome. Figure 1 shows the chromosome maps of DSY562 as compared to CBS138 as well as a circular map to highlight major translocations between the reference and the DSY562 genomes. For practical reasons, the naming of chromosomes (A to M) was kept as proposed initially for CBS138. The data highlight several chromosomal rearrangements between the two isolates. While Chr A, B, E, G, H, and K kept similar structures between both strains, other chromosomes underwent several modifications. Chr C, D, J, and M in DSY562 underwent exchanges from CBS138 chromosomes essentially at chromosomal extremities. Parts of Chr L and I were added to Chr F (which was renamed as Chr F-L-I) in DSY562, thus yielding the largest chromosome in this species (1.59 Mb). Chr D and L were joined to the shortened Chr I (which was renamed as Chr I-L-D). The left arms of Chr L and C were inverted as compared to CBS138. Remarkably, the separate DSY565 assembly yielded the same chromosome arrangement as DSY562, with the exception that Chr J was not completely assembled in DSY565. This chromosome was still split in two parts (Chr J1 and J2; see Table 4 and Figure S1) in DSY565. A CHEF was performed with strains CBS138 and DSY562/DSY565 to compare these assemblies with physical chromosome separations. Remarkably, size predictions from the assemblies corresponded well to physical separations (see Figure 2). Red arrows shown in Figure 2 correspond to size conservation of Chr A, B, G, H, and J between the two strains, which is consistent with predictions. Chr C, D, E, and F-L-I were shifted to higher sizes (yellow color) by CHEF in DSY562, which is also consistent with assembly predictions. Chr M and I-L-D exhibited decreased sizes (blue color) as compared to CBS138, which is also predicted from assemblies. Chr K from DSY562 showed a discrepancy from the expected patterns from CBS138, however this result may be due to intrinsic chromosome size variations observed by others for this type of strain (Bader *et al.* 2012).

**Table 4:** DSY562 and DSY565 annotation features

DSY562						DSY565						CBS138				
Chr	CDS	tRNA	rRNA	Introns	Confirmed Introns	Chr	CDS	tRNA	rRNA	Introns	Confirmed Introns	Chr	CDS	tRNA	rRNA	Introns
A	201	9		10	7	A	201	9		10	6	A	205	9		9
B	218	9		4	3	B	218	9		4	3	B	220	9		4
C	242	7		4	1	C	240	7		4	1	C	235	7		7
DL	300	18		7	6	DL	300	18		7	7	D	288	18		6
E	279	9		8	5	E	279	9		8	5	E	282	9		10
F-L-I	661	29		19	15	F-L-I	661	8		19	15	F	387	15		12
G	438	18		18	16	G	438	29		18	17	G	440	18		18
H	470	14		11	9	H	470	18		11	9	H	466	14		12
I-L-D	278	18		2	2	I-L-D	278	14		2	2	I	470	26		6
J	522	17		18	18	J1	454	18		18	16	J	523	17		19
						J2	67	9		1	1			22		
K	562	22		16	13	K	561	22		16	14	K	564	25		17
L	484	19	2	8	7	L	485	19	2	8	7	L	582	18	4	11
M	623	18	3	16	12	M	622	18	3	16	12	M	620			16
Mitochondria	11	23	2	3	—	Mitochondria	11	23	2	3	—	Mitochondria	11	23	2	3
Other contigs	5		2			Other contigs	13		1							
Total	5294	230	9	144	114		5298	230	8	144	115		5293	230	6	150

With these data, we first compared the DSY562 PacBio-assembled data (DSY562-HGAP3) with the CBS138 published genome (CGD, version_s02-m04-r02). The DSY562-HGAP3 genome was first annotated with the tools available in the software Geneious (version 9.1.8, “live-annotation” tool) with a threshold level of 95% between comparisons. DSY562-HGAP3 was also further annotated on the basis of a recently published annotation using CBS138 and RNAseq data (Linde *et al.* 2015). As observed from Table 4, the number of deduced CDS (locus tag prefix B1J91) in DSY562 was 5294, which was near to the 5293 predicted CDS from CBS138. The DSY565 genome was next annotated on the basis of DSY562 annotations with the same Geneious tool and yielded 5298 CDS (locus tag prefix B1J92). Table 4 indicates that the number of CDS in mitochondrial DNA and numbers of tRNA were identical between strains. rDNAs were identified in DSY562 and DSY565, and were located at the telomeric ends of Chr L and M-J as in CBS138.

**Figure 3:**
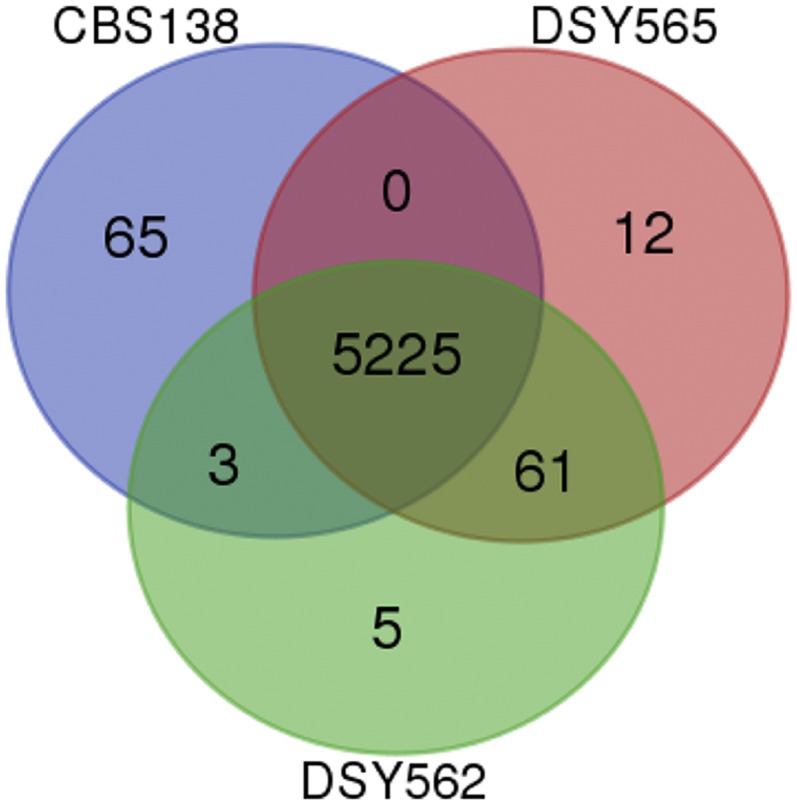
Venn diagram representation of genes shared between DSY562, DSY565, and CBS138. The diagram was generated with a Web tool available at http://bioinformatics.psb.ugent.be/webtools/Venn/.

The similarity between the total number of CDS in the three strains (5293–5298 CDS) obscures, however, the following divergence: when comparing the CDS of each isolate, only a total of 5225 CDS were shared between them. 65 CDS from CBS138 were not detected in DSY562 and DSY565 (Figure 3 and File S3). 78 CDS from DSY562 and DSY565 were not found in CBS138. From these 78 CDS, 61 were common to DSY562 and DSY565; thus highlighting unique CDS for each of the strains. These above-mentioned results suggest that several selection events resulted in the loss and gain of several genes between these strains. When inspecting the identity of these 61 genes, we found that most of the genes gained in DSY562 and DSY565 as compared to CBS138 belonged to adhesin-like genes (34). Nine genes originating from DSY562 and DSY565 (named here Tkp5-1 to -9) were most similar to the Tkp5 protein from *Vanderwaltozyma polyspora*. This type of protein encodes elements of Ty-like retrotransposons (see *Discussion*). Other common genes between DSY strains underwent duplication events, including B1J91/B1J92_D02794g (endoplasmic reticulum membrane protein), B1J91/B1J92_M05115g (a putative retrotransposon protein), B1J91/B1J92_H04257g, and B1J91/B1J92_H04279g (encoding copper metallothionein MT-II and MT-IIB in DSY562 and DSY565 respectively). Interestingly, B1J91/B1J92_H04257g and B1J91/B1J92_H04279g were found in four tandem repeats on Chr H as opposed to CBS138, in which only one repeat is found. The remaining 10 genes out of the 61 common to DSY562 and DSY565 were ranked as pseudogenes in the current CGD (some of which are also described as adhesin-like genes), thus explaining their absence from the CBS genome used in our analysis. Genes that were CBS138 specific (65) included, among others, adhesin-like genes (6), CAGL0B01243g (MATα1), and CAGL0G07175g (RNA binding protein with RNA-directed DNA polymerase activity). Notably, MATα1 is still present in DSY562 and DSY565 but as another CDS (B1J91/B1J92_B00242g). Most of the remaining CBS138-specific genes were of unknown function (48) (see details on File S3).

At the level of single nucleotides, large differences were observed between DSY562 and CBS138 (Table 5). Nuclear and mitochondrial genomes diverged by 0.7% between the two strains. These data indicate extensive divergence between the two genomes, however they are in general agreement with the analysis deduced from other *C. glabrata* genomes (Singh-Babak *et al.* 2012). The high number of nonsynonymous SNPs (11,014) underscores a large number of amino acid substitutions. A high number of SNPs occurred in genes encoding adhesins, and the following section will discuss the resolution of this important gene category. A few genes underwent frameshifts resulting in protein truncations. For example, B1J91/B1J92_D00154g (*AQY1*, role in ion transport) was truncated in DSY562 and DSY565 (CDS length of 459 bp) as compared to CBS138 (CDS length of 873 bp). Interestingly, another homolog is present in DSY562 and DSY565 (B1J91/B1J92_A01221g), thus likely compensating for the probable loss of function of B1J91/B1J92_D00154g. B1J91/B1J92_C02343g (*ARB1*: a protein with ATPase activity and a role in cellular response to drugs, a ribosomal small subunit exported from the nucleus and cytosol), which contains an intron in CBS138, lacks a reading frame in DSY562 and DSY565. Removal of the intron in this gene restores a truncated CDS as compared to CBS138. B1J91/ B1J92_E01309g (*EKI1*: choline kinase activity, a role in the phosphatidylcholine biosynthetic process) exhibited an N-terminal deletion due to the use of other intron splicing as compared to the CBS138 homolog. B1J91/B1J92_M05687g (*NPY1*: has NAD+ diphosphatase activity and a role in the NADH metabolic process), B1J91/B1J92_G03487g (*TMN3*: has a role in cellular copper ion homeostasis), B1J91/B1J92_I10725g (YGR122W: has a role in negative regulation of transcription from the RNA polymerase II promoter), B1J91/B1J92_E01155g (*RPA14*: RNA polymerase I activity), and B1J91/B1J92_H06017g (*FLR1*: a multidrug transporter of the major facilitator superfamily) exhibit N-terminal truncated proteins as compared to CBS138. Whether the identified protein truncations may result in deficient functioning remains to be established. We believe that these changes do not reflect PacBio errors since (i) the DSY562 and DSY565 assemblies were produced independently to produce the same changes as compared to CBS138, and (ii) Sanger sequencing verifications confirmed the absence of PacBio errors [see example of B1J91/B1J92_H06017g (*FLR1*) sequencing in Figure S2]. We also noticed that some previously annotated tandem CDS in CBS138 were now forming a single CDS in DSY562. For example, B1J91/B1J92_I02816g and B1J91/B1J92_I02838g (*AZF1*: asparagine-rich zinc finger protein) were now generating a single CDS (B1J91/B1J92_I02838g) in DSY562. B1J91/B1J92_E03421g and B1J91/B1J92_E03432g (close homologs of *Saccharomyces cerevisiae CDA1* and *CDA2* genes; chitin deacetylases) were now forming a single CDS (B1J91/B1J92_E03421g). It is interesting to observe that there are ∼370000 nt differences between the CBS138 and DSY562 genomes (the latter having the largest genome), thus highlighting that additional/novel genes may be present in the DSY strains (see below). As a last step in these comparisons, we analyzed the presence of introns in strains DSY562 and DSY565 as compared to CBS138. This was performed first by the transfer of annotations from the recently updated CBS138 genome. We next used RNAseq data from both DSY562 and DSY565 transcriptomes to detect spliced RNAs. Table 5 shows that DSY562 and DSY565 genomes possessed at least 144 intron-containing genes, which is close to the number identified in CBS130 (150). RNAseq data from DSY562 and DSY565 showed that 114 and 115 splice sites, respectively, coincided with the predicted splice sites (File S4).

**Table 5:** Whole genome differences between DSY562 and CBS138

	CBS138 *vs.* DSY562				
	Nonsynonymous SNP	Synonymous SNP	In/Outside SNPs Indels	Indels	Total Percentage SNP-Indel *vs.* CBS
Total	11,014	25,845	86,915	5353	
Percentage *vs.* CBS	0.09	0.21	0.7	0.04	0.7
Mitochondrial	34	11	140	57	
Percentage *vs.* CBS	0.17	0.055	0.7	0.285	0.7

### The cluster of adhesins in DSY562

**Figure 4:**
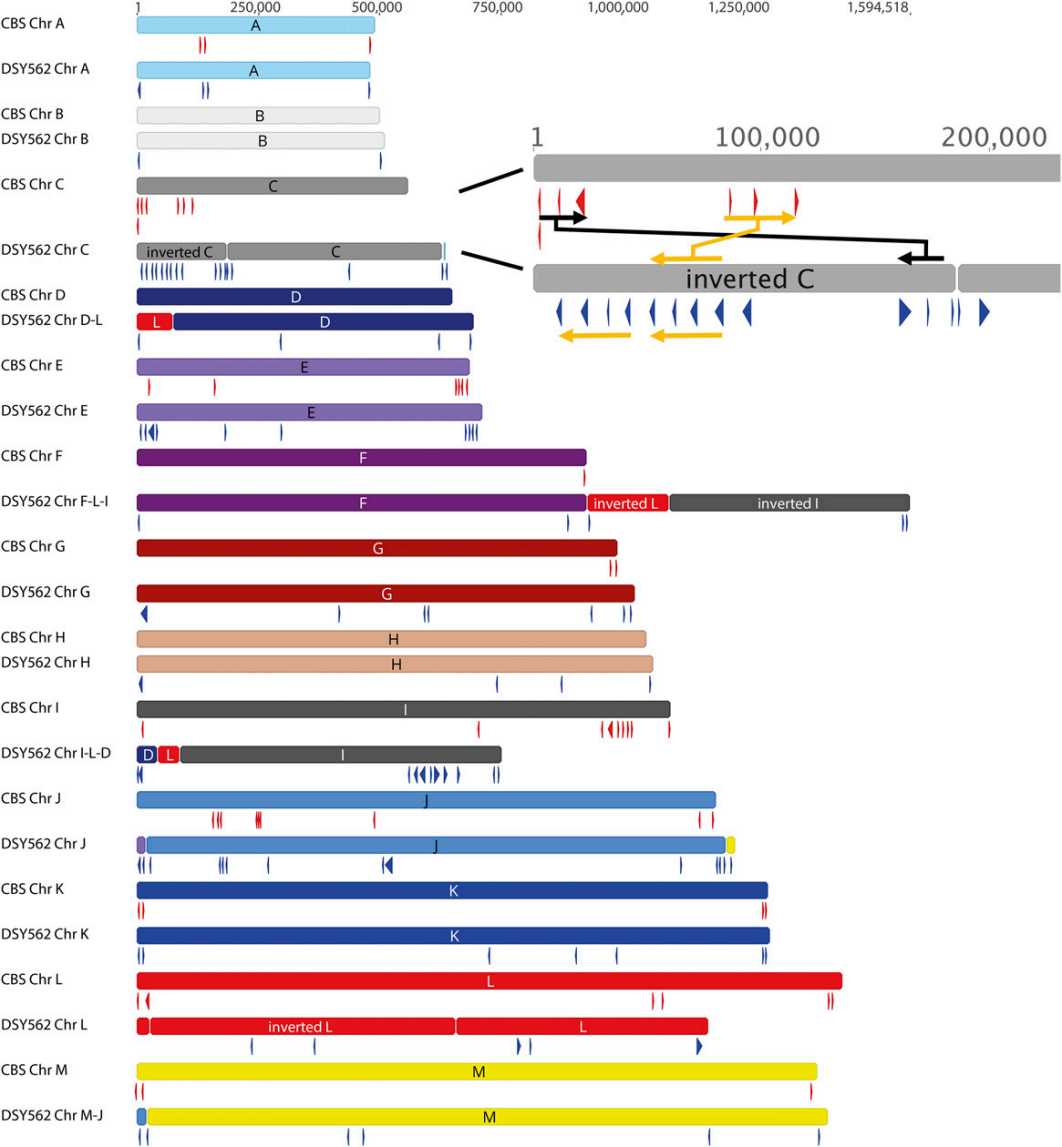
Arrangement of adhesin-like genes in *C. glabrata* CBS138 and DSY562. The position of adhesins is shown by red and blue →’s in CBS138 and DSY562, respectively. Chr C ends of DSY562 and CBS138 were enlarged to highlight the positioning of adhesins in the two genomes. Black →’s indicate the position of adhesins in CBS138 which were inversely located in the Chr C arm of DSY562. A group of three tandemly arranged adhesins in CBS138 (orange →) is increased by one member in DSY562. This group of four adhesins is tandemly arranged with a second group of four adhesins.

**Figure 5:**
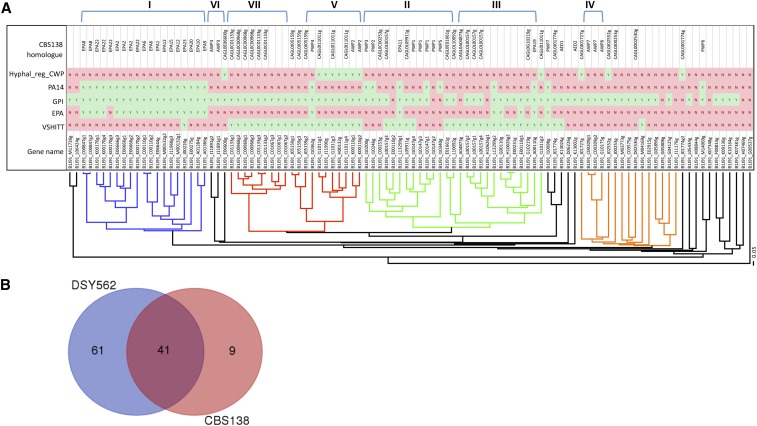
Adhesins in DSY562. (A) Phylogenetic tree of DSY562 adhesin-like genes. Each gene is labeled with conserved PFAM functional domains (see *Materials and Methods*). Adhesin clusters (I–VII) were those reported in de Groot *et al.* (2008). (B) Difference between DSY562 and CBS138 adhesins.

*C. glabrata* is known for its extensive repertoire of adhesins. The genes encoding adhesins often contain repeated motifs and are mostly localized at chromosome extremities. These two characteristics make it challenging to establish a detailed and complete listing of adhesins from *C. glabrata* genomes. Here we have used PacBio genome assembly approaches to address these challenges and we used a systematic analysis (described in *Materials and Methods*) to identify adhesin-like genes in DSY562 and DS565. Briefly, all DSY562 CDS were screened with the help of the software FungalRV, which helps to predict the occurrence of adhesin-like proteins in fungal genomes (Chaudhuri *et al.* 2011). We next analyzed the list of potential adhesins for the occurrence of PFAM domains and the presence of specific signatures, including (i) the presence of signal peptides and absence of internal transmembrane domains, (ii) the presence of a consensus sequence for GPI anchor, (iii) the presence of AWP motifs (de Groot *et al.* 2013), (iv) the presence of motifs typical for the class of EPA proteins following Diderrich *et al.* (2015) and Gabaldón *et al.* (2013), (v) the presence of PIR motifs, and lastly (vi) the occurrence of hyphally regulated cell wall proteins (Hyphal_reg_CWP, IPR021031). For practical reasons and for comparison purposes with CBS138, we used the same CAGL0 gene naming that is currently accepted for CBS138. When several highly similar proteins were identified in DSY562, we add a numerical suffix to them (*i.e.*, 1, 2, *etc*.). According to our analysis, we proposed a list of 101 adhesin-like proteins in DSY562 (File S5 and File S6), which well exceeds the number of deduced adhesins in CBS138 (66) according to de Groot *et al.* (2013). Figure 4 shows the genomic localization of adhesins detected in DSY562 as compared to CBS138. Notably, the repertoire of adhesins in DSY565 is very similar to DSY562 (Figure S1, see below). ∼50% of the adhesin genes were located in DSY562, near to the telomeric ends (region of 50 kb down to chromosome ends) of the chromosomes. One example of the expansion of adhesins in DSY562 is given by the end of Chr C (Figure 4). The size difference between Chr C between DSY562 and CBS138 may be explained by the tandem arrangement of a group of four adhesins. The DSY562 potential adhesins were aligned and a phylogenetic tree was assembled (Figure 5). This tree was merged with conserved domain features and with CBS138 protein homologs to, on the one hand, help distinguish between these proteins and, on the other hand, to better delineate extensions of protein families in DSY562. Several protein clusters (highlighted by colors in Figure 5) were observed. Members of the EPA family (19) were well discerned in this tree (blue color of Figure 5) and harbor typical signatures of EPA adhesins, including a PA14 domain as well as EPA-specific domains (see details in *Material and Methods*), with the exception of B1J91_K00170g4. Duplication of specific EPAs occurred in DSY562 for *EPA2*, *EPA6*, *EPA8*, *EPA12*, and *EPA22*. The largest cluster of proteins contained VSHITT-motif repeats and PA14 motifs, however, they lacked EPA signatures (green color of Figure 5). CBS138 PWP homologs (PWP1, -2, -3, and -5) were found in this cluster. VSHITT-motif repeats were also found in another cluster (red color of Figure 5) that essentially lacked PA14 motifs. The largest extension occurred for adhesin-like proteins with VSHITT repeats that include members of the PWP and AWP family (Figure 5). Another well-distinguished cluster lacked both VSHITT and PA14 motifs and contained CBS138 AWP homologs (AWP6 and -7). Only one gene (B1J91_M11726g) contained a PIR motif and was extensively unrelated to other genes of the phylogenetic tree. The remaining proteins could not be assembled in clusters with common features. Taken together, the data suggest that 61 novel adhesin-like proteins were assigned to DSY562, while 40 remained in common with CBS138 (Figure 5, only 50 adhesin-like proteins without frameshifts were taken into account in these calculations). The majority of the novel proteins exhibited VSHITT domains.

### Genome comparisons between DSY562 and DSY565

**Figure 6:**
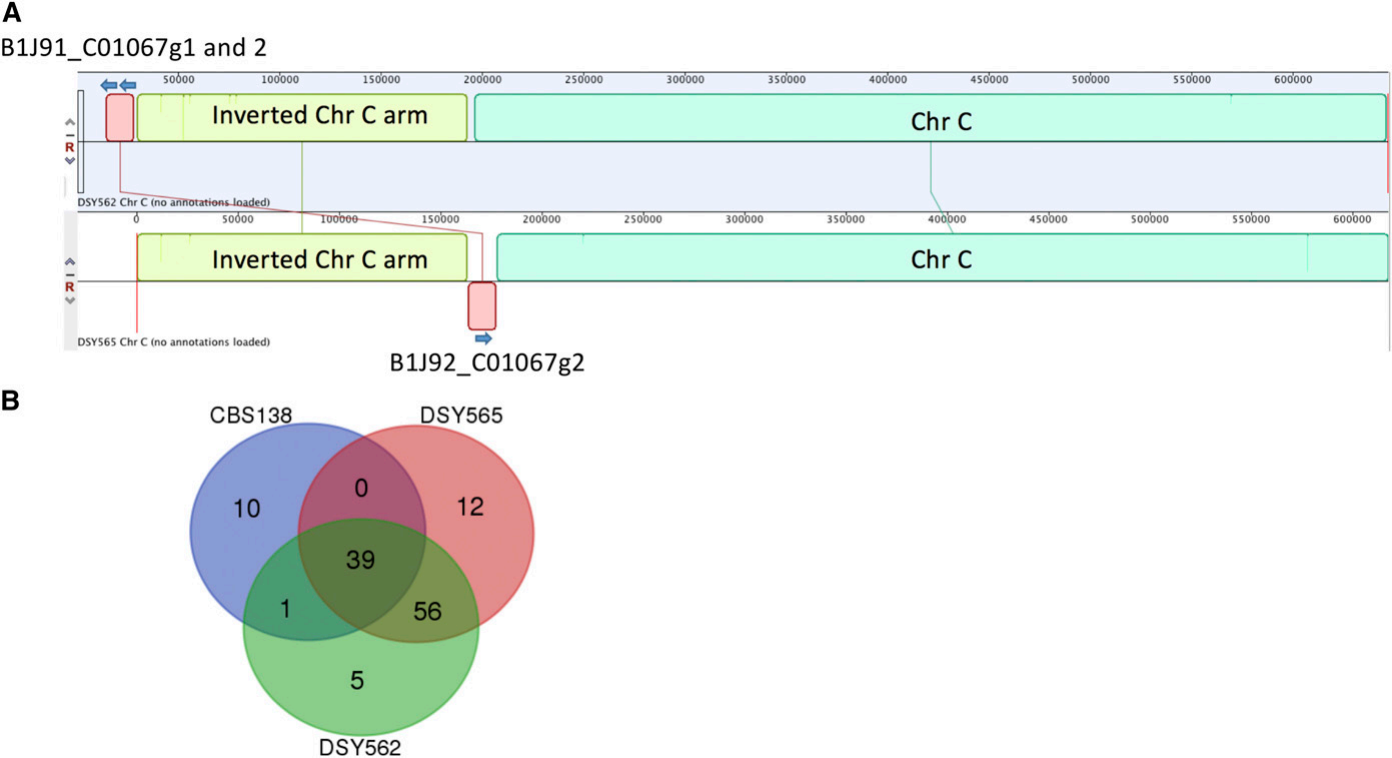
Adhesin distribution between CBS138 and DSY strains. (A) Divergent position of adhesins between DSY565 and DSY562. The map was created with the Mauve plug in available in the Geneious software. (B) Venn diagram representation of adhesins shared between CBS138, DSY562, and DSY565 isolates. Venn diagram data can be found in File S6.

As we mentioned earlier, DSY5652 and DSY565 are two highly related isolates that were obtained from a patient treated with fluconazole for oropharyngeal candidiasis. DSY565 was isolated ∼50 d after initiation of fluconazole treatment and was azole resistant. We reported that azole resistance was due to a mutation in the transcription factor *CgPDR1*. This mutation was contributing to an increase of virulence as compared to a parental strain and was measured in different animal models (Ferrari *et al.* 2009; Vale-Silva *et al.* 2016). Given that virulence traits may be multifactorial, we reasoned that other mutations may contribute to this phenotype. Direct genome comparisons with PacBio-generated data may provide some cues into this hypothesis. We first annotated the DSY562 genome to a high resolution as described above and used this basis as a tool to annotate the DSY565 genome. The use of CBS138 as an annotation basis provided similar results (data not shown). Table 2, Table 3, and Table 4 compare several features between DSY562 and DSY565. At the genomic level, a few changes occurred. While 101 adhesin-like genes were identified in DSY562, 107 adhesin-like genes were found in DSY565 (Figure S1). 95 adhesins were shared between both isolates (Figure 6) and the discrepancies between the two genomes were principally due to the presence of adhesin-like genes in small contigs, which could not be mapped to the chromosomes (Figure S1). 12 and three adhesin-like and unique genes were identified in the small contigs of DSY565 and DSY562, respectively. In addition, DSY562 possesses one gene (B1J91_E00110g1) located at the extremity of Chr J that is not present in DSY565 (Chr J1). This may be due to incomplete assembly of Chr J1 in this isolate. One other specific change between DSY562 and DSY565 occurred at the left arm of Chr C. A size difference of ∼29 kb exists at the left arm of Chr C between DSY562 and DSY565 (Figure S1). While a tandem repeat of B1J91_C01067g is present in DSY562 at the Chr C extremity, only one copy remained in DSY565. However, this gene is now situated between the adhesins B1J92_C00968g1 and B1J91_C01133g1 of DSY565 (Figure 6). One remaining copy of this specific adhesin (B1J92_C01067g1) is situated in the small contig unitig16_17kb, which suggests that this small contig may be assigned to the missing nucleotides of the DSY565 Chr C left arm.

Another major difference between DSY562 and DSY565 is that DSY565 lacks the retrotransposon Tkp5-7 that is situated at the right arm of Chr M in DSY562. Interestingly, DSY565 has retained a single long terminal repeat (LTR) at this locus as a trace of the presence of the retrotransposon. A PCR-based approach confirmed the absence of Tkp5-7 in DSY565 while leaving the LTR signature (see Figure S3).

At the single nucleotide level, differences between DSY562 and DSY565 include synonymous and nonsynonymous SNP variations as well as indels. Table 6 gives an overview of the observed differences. First we noticed that most of the indels in these comparisons were in homopolymeric sequences, which are long stretches (10–30) of identical nucleotides (108 in total). Since it is known that PacBio can yield these differences, we undertook systematic verifications of these sequences by separate Sanger sequencing of small PCR fragments comprising these putative indels. Only eight of these indels were confirmed by this approach (95 out of 108 were corrected; see Table 6 and File S7). Other indels in nonhomopolymeric sequences (14 indels − 8 indels in homopolymeric sequences = 6) were identified by genome comparisons between DSY562 and DSY565. Their presence was confirmed by separate genome alignment with Illumina reads obtained in the early stage of the project (data not shown). We also identified a number of indels within coding regions that were also nonhomopolymeric. These variations between the two strains ranged from three to a larger number of nucleotides, however only a few of them created frameshifts (see Table 8).

**Table 6:** Nucleotide variations between DSY562 and DSY565

Chr name	DSY562 *vs.* DSY565					
	Nonsynonymous SNP	Indels in CDS	Synonymous SNP	In/Outside SNP	Indels	Corrected Indels in Homopolymers
Chr A	2	0	0	3	0	4
Chr B	0	0	0	1	0	1
Chr C	1	1	0	3	1	4
Chr D-L	3	1	1	4	1	7
Chr E	2	2	2	4	0	8
Chr F-L-I	1	1	3	7	3 (2)*^a^*	17
Chr G	0	0	1	1	2 (1)*^a^*	9
Chr H	1	0	0	2	3	4
Chr I-L-D	3	3	1	6	0	4
Chr J1	0	1	2	5	3	3
Chr J2	0	1	1	0	0	1
Chr K	1	0	0	1	0	10
Chr L	0	1	0	9	0	4
Chr M-J	3	0	0	6	1	16
Mitochondrial DNA	0	0	0	0	0	3
Total	17	11	11	52	14	95

a Number in parentheses indicates the number of single nucleotide indels.

A total of 17 nonsynonymous SNPs were identified between both genomes (Table 7). As expected, the transcription factor *CgPDR1* harbored the L280F substitution in DSY565. This mutation is known to be sufficient to mediate azole resistance in *C. glabrata* (Ferrari *et al.* 2009). The other nonsynonymous SNPs were located in genes not known to be associated with drug resistance. Related with transcription was the identification of a nonsynonymous SNP in B1J91_L0L01001g (*NOT3*: subunit of CCR4-NOT, a global transcriptional regulator). Three other nonsynonymous SNPs were located in genes involved in rRNA metabolism and ribosome biogenesis: B1J91_H02057g (*GAR1*: nucleolar rRNA processing protein), B1J91_M00946g (YLR435w: a protein with a potential role in pre-rRNA processing), and B1J91_M08954g (YOR019w: a protein of unknown function; may interact with ribosomes). We noticed several genes containing nonsynonymous SNPs that annotated with transporter functions, including B1J91_A01804g (HXT3/HXT1: hexose transporter), B1J91_D02442g (*LEM3*: translocation of phospholipids and alkylphosphocholine drugs), B1J91_K04565g (YGR065c: vitamin H transporter), and B1J91_M13233g (*MNR2*: vacuolar membrane protein; putative magnesium transporter). Two other nonsynonymous SNPs were situated in adhesin genes (B1J91_C00968g3 and B1J91_C00209g2). The question of whether or not these mutations result from specific selection pressure from the host or from drug exposure cannot be answered yet; but either is possible. Besides these nonsynonymous SNPs, larger deletions an insertions were identified in coding sequences (Table 8). The majority of these deletions/insertions (7 out of 11) were observed in adhesin-like genes. We cannot presently exclude that these changes are the results of PacBio read mis-assemblies. Some of the affected CDS are located near to chromosome ends, which are known to be recalcitrant to correct assembly and include repetitive sequences. It is likely that recombination events in these regions may generate variations which could result in a beneficial phenotype for the fungus.

**Table 7:** Nonsynonymous SNPs between DSY562 and DSY565

Chr	Locus_tag*^a^*	Gene Name*^b^*	Nucleotide Position*^c^*	Nucleotides Change	Amino Acid Change	Function
Chr A	B1J91_A01804g	HXT3/HXT1	185747	C → G	V560 → L	Hexose transporter
Chr A	B1J91_A00451g	PDR1	53729	G → C	L280 → F	Transcription factor
Chr C	B1J91_C00968g3	CAGL0C00968g	41110	GCA → TTG	A993 → N	Putative adhesin-like cell wall protein
Chr D-L	B1J91_D02442g	LEM3	406506	T → C	V81 → A	Translocation of phospholipids and alkylphosphocholine drugs
Chr D-L	B1J91_L00429g	GSD2	648612	C → T	L633 → F	Glycine dehydrogenase
Chr D-L	B1J91_D03762g	GIC2	274967	G → T	T280 → K	CDC42 GTPase-binding protein
Chr E	B1J91_E01441g	SAN1	160147	C → T	P575 → S	Ubiquitin-protein ligase
Chr E	B1J91_C00209g2	AWP7	20343	A → G	I71 → T	Putative adhesin-like cell wall protein
Chr F-L-I	B1J91_F05709g	ATC1	584210	A → C	268E → D	Nuclear protein; possibly involved in regulation of cation stress responses
Chr H	B1J91_H02057g	GAR1	873919	C → T	92T → I	Nucleolar rRNA processing protein
Chr I-L-D	B1J91_L01001g	NOT3	85356	T → A	E118 → D	Subunit of CCR4-NOT global transcriptional regulator
Chr I-L-D	B1J91_I07601g	COQ3	333430	G → A	P171 → L	Mitochondrial ubiquinone biosynthesis
Chr I-L-D	B1J91_I08965g	YSC84	471495	C → T	G75 → R	Actin-binding protein
Chr K	B1J91_K04565g	YGR065c	434920	A → G	I218 → M	Vitamin H transporter
Chr M-J	B1J91_M08954g	YOR019w	522442	C → G	Q546 → E	Protein of unknown function; may interact with ribosomes
Chr M-J	B1J91_M00946g	YLR435w	1307238	C → A	D101 → Y	Protein with a potential role in pre-rRNA processing
Chr M-J	B1J91_M13233g	MNR2	105774	C → T	243R → H	Vacuolar membrane protein; putative magnesium transporter

a DSY562 locus_tag is given here.

b Gene name is inferred from the CBS138 annotations.

c Nucleotide position is from the DSY562 genome.

**Table 8:** Deletions/insertions in CDS between DSY562 and DSY565

Chr	Locus_tag	Gene Name	Nucleotide Position	Nucleotide Change	Type of Change*^a^*	Amino Acid Change	Function
Chr C	B1J91_C01067g3	CAGL0C01067g	52087	AGCTAGGAGCAG	Insertion	SAPSS →766	Putative adhesin-like cell wall protein (insertion in DSY562)
Chr D-L	B1J91_L00157g4	CAGL0L00157	4453	591 bp	Deletion	V1392 → 198 aa	Putative adhesin-like cell wall protein
Chr E	B1J91_E02783g	*SLA1*	287558	TGCTCA	Deletion	1040 → AQ	Cytoskeletal protein binding protein
Chr E	B1J91_E00561g	*TUP1*	67152	CAA	Deletion	100 → Q	Transcriptional repressor
Chr F-L-I	B1J91_I05082g	YBL051c	1114101	(CAA)9 → (CAA)10	Deletion	177 → Q	Response to DNA damage
Chr I-L-D	B1J91_E06666g2	CAGL0E06666g	749114	40 bp	Deletion	N895 → fs	Putative adhesin-like cell wall protein
Chr I-L-D	B1J91_I07293g	CAGL0I07293g	307101	135 bp	Deletion	N669 → 198 aa	Putative adhesin-like cell wall protein
Chr I-L-D	B1J91_I10147g1	CAGL0I10147g	585641	303 bp	Insertion	Y3995 → fs	Putative adhesin-like cell wall protein
Chr J1	B1J91_J01800g	CAGL0J01800g	5653	278 bp	Deletion	P399 → 92 aa	Putative adhesin-like cell wall protein
Chr J2	B1J91_E00110g2	CAGL0E00110g2	11438	299 bp	Deletion	Y2078 → 2750 aa*^b^*	Putative adhesin-like cell wall protein
Chr L	B1J91_L09251g	CAGL0L09251g	723722	(TCT)12 → (TCT)13	Deletion	155 → E	Positive regulation of transcription from RNA polymerase II promoter

a Change in DSY562 *vs.* DSY565.

b Changes reflect that B1J91_E00110g2 was extended in DSY565 by 2750 aa, which restores a single CDS from B1J91_E00110g1 and B1J91_E00110g2.

## Discussion

We described here the genomes of two *C. glabrata* sequence isolates, DSY562 and DSY565, originating from an HIV-positive patient with oropharyngeal candidiasis. The close relationship between these two isolates reported earlier (Sanglard *et al.* 1999) is confirmed here by the high level of similarity between the sequenced genomes. We showed earlier that DSY565 contained a GOF mutation in *PDR1* (L280F), contributing to azole resistance but also enhancing virulence in animal models. *PDR1* GOF can modulate the expression of the adhesin *EPA1* and this effect results in increased adherence to host tissues. Enhanced virulence due to *PDR1* in DSY565 may also be due to other factors. On the other hand, we observed that DSY562, the parent of DSY565, was intrinsically highly adherent to host cells, even with the lowest *EPA1* expression levels as compared to other tested strains. This intrinsically high adherence capacity was observed with a *PDR1* wild-type allele (Vale-Silva *et al.* 2016). Interestingly, a separate study demonstrated that DSY562 was a *C. glabrata* isolate with the highest capacity among those tested to bind *C. albicans* hyphae (Tati *et al.* 2016). Binding of *C. glabrata* involved the *C. albicans* Als3 adhesin. All these data suggested the potential presence of other adhesins in the genetic background of DSY562 and its related strain DSY565. Given these reasons, we aimed here to obtain the two *C. glabrata* genomes with a high resolution to identify all possible differences between the two genomes and to determine all possible adhesin and adhesin-like genes in the two genomes.

At the genomic level, DSY562 and DSY565 underwent several major chromosomal rearrangements. No data are currently available on PacBio-assembled *C. glabrata* genomes of sequential isolates, and it is therefore difficult to compare our results with other studies. One recent short report described the genomes of three pairs of *C. glabrata* isolates from patients with candidemia (Håvelsrud and Gaustad 2017). The genomes were assembled *de novo* using Illumina reads, thus resulting in different genome outputs (119–186 scaffolds). Comparison between the DSY genomes and the three published pairs using the same chromosomal fragments highlighted divergences as high as those between DSY isolates and CBS138. This underlines the high genetic diversity existing between *C. glabrata* isolates of different origins which has been observed by others using different approaches. One other recent study also applied Illumina-based *de novo* assembly of 33 genomes (Carreté *et al.* 2017) which identified 17 different chromosome patterns. Some patterns are similar to those observed in DSY562 and DSY565. While Chr D-L and Chr L inversions, as well as Chr I-L-D are among the common patterns, Chr F-L-I and Chr C inversions seem unique to DSY562 and DSY565. Another separate study established the genome of a *C. glabrata* isolate (CCTCC M202019) which is an industrial yeast strain widely used to produce α-oxocarboxylic acid (Xu *et al.* 2016). Genome data were established on the basis of a 150-bp, pair-end library sequenced by Illumina approaches and 111 contigs and 74 scaffolds were generated. Interestingly, this *C. glabrata* isolate was remarkably closely related to CBS138 (205 SNP differences) (Xu *et al.* 2016).

Taken together, these studies underline the high genetic diversity existing between *C. glabrata* isolates of different origins. It is likely that future studies will reveal additional patterns. In proportion to the size of their genomes, the number of changes between the DSY strains is rather small. We noticed only 17 nonsynonymous changes between these isolates (Table 7). Besides the known L280F change in *PDR1*, several other proteins with diverse functions were affected. The role played by these alterations is not clear at this time. One might speculate that they could contribute to the fitness of DSY565 or that they might be accidental. Other larger changes were detected between both strains (Table 8) and they occurred mainly in adhesin-like genes. This could reflect some adaptation due to host selection pressure.

It has been reported earlier that the mismatch-repair gene *MSH2* can modulate drug resistance when its activity is compromised by specific SNPs, giving rise to a hyper-mutator phenotype (Healey *et al.* 2016b). Interestingly, DSY562 and DSY565 carry a V239L nonsynonymous mutation in *MSH2*, which is known to favor this hyper-mutator phenotype (Healey *et al.* 2016b). One might expect that this phenotype could generate genetic diversity within the context of host conditions. DSY565 was isolated with a time-lapse of 50 d in the oral cavity of a patient as compared to DSY562. Within this elapsed time, the *MSH2* defect may lead to mutation accumulations. In a study probing the accumulation of mutations due to *MSH2* defects in *S. cerevisiae* over 170 generations *in vitro*, mutation rates (mutations per bp per generation) were in the range of 5–9 × 10^−8^, which corresponded to 8–16 single base pair substitutions and 110–180 deletions/insertions in the haploid *S. cerevisiae* genome (Lang *et al.* 2013). The number of generations that occurred within the time-lapse of DSY562 and DSY565 isolation may correspond to at least 250–500 generations, when taking into account 5–10 generations/d according to published data (Forche *et al.* 2009; Roetzer *et al.* 2011). Taking into account the data of Table 7 and Table 8, the number of SNPs and indels between DSY562 and DSY565 are in agreement with numbers reported in *S. cerevisiae*. One may expect that in *C. glabrata* the *MSH2* defect may generate random mutations with a negative effect on *in vivo* fitness. The host conditions may serve as a selection pressure to eliminate progeny with fitness costs. We only sampled successful progenitors from the clinic, which of course strongly reduces the diversity of isolates for further analysis. This underpins that genetic diversity by the *MSH2* defect may be much higher than observed from the collected clinical strains. The current literature on the effect of DNA mismatch-repair defects also highlighted that the occurrence of mutations is not random: DNA regions with a greater density of repeats are more mutable in mismatch-repair defective cells (Ma *et al.* 2012; Lang *et al.* 2013). In the context of adhesins, which are enriched in repetitive elements, *MSH2* defects may perhaps contribute to increase the diversity of this gene family over generations. It has been observed that 44–50% of investigated isolates (*n* = 625) were carrying *MSH2* defects which thus indicates that the *MSH2* defect may confer some selective advantage in *C. glabrata* (Dellière *et al.* 2016; Healey *et al.* 2016a,b). In agreement with our hypothesis, it is interesting to mention that CBS138 and CCTCC M202019 do not exhibit *MSH2* defects and they contain a limited repertoire of adhesins as compared to DSY562/DSY565.

Besides the above-mentioned SNPs, the DSY562 and DSY565 genomes harbored transposon-like genes not reported previously in *C. glabrata*. These Ty-like retrotransposons genes (named here Tkp5-1 to -9) were most similar to the Tkp5 protein from *V. polyspora*, a yeast representing the postwhole-genome duplication lineage most divergent from *S. cerevisiae* (Scannell *et al.* 2007). We observed that, while Tpk5-2 and Tpk5-5 to -9 encoded the same proteins, Tpk5-3 and -4 were different (66% identity) but still similar to the Tkp5 protein from *V. polyspora*. Tpk5-1 shared the C-terminal ends of Tpk5-3 and -4. As expected from retrotransposons, Tpk5-2 and Tpk5-5 to -9 were flanked by the same LTRs, while Tpk5-3 and -4 were flanked by other distinct LTRs. Curiously, Tpk5-1 was located next to B1J91/B1J92_M05115g on Chr G, which is described as similar to Ty5-6, a *S. paradoxus* retrotransposon. Both genes are flanked by the same LTR. Taken together, these features suggest that DSY562 and DSY565 have acquired retrotransposons from different sources and that these strains have undergone several transposition events. Notably, DSY565 did not retain Tkp5-7, which is otherwise situated at the right arm of Chr M in DSY562. Given that a single LTR remained at this location in DSY565, this suggests an active transposition system. This retrotransposon was not identified in another location in DSY565.

We determined the presence of >100 adhesin-like genes in both DSY strains, which was not yet anticipated from other genome-wide studies. This number exceeds by far the numbers published for CBS138 (50–66, depending on the criteria used for selection) and therefore suggests that an expansion of this gene family occurred in our isolates. A genome report on a separate *C. glabrata* isolate closely related to CBS138 identified 49 adhesin-like genes (Xu *et al.* 2016). Since no equivalent *C. glabrata* genome assembly has been yet published using a PacBio approach, we still cannot confirm whether or not the investigated isolates constitute a unique case or that a high repertoire of adhesin genes (>100 genes) is shared in several *C. glabrata* clades. The number of identified adhesins between both isolates differs substantially (101 in DSY562 *vs.* 107 in DSY565). It is unlikely that this discrepancy could reflect an expansion of adhesins in DSY565 as compared to DSY562 in such a short time-lapse between sampling of isolates. We rather attribute this difference to technical issues during the assembly of PacBio reads. These reads produced small contigs, as illustrated in Figure S1, which are enriched in adhesin genes. These genes probably form part of chromosomal ends, but remain presently unassembled to major contigs.

To help distinguish the adhesins in DSY562 and DSY565, we used a combination of hierarchical clustering and mapping of PFAM and functional domains. With this approach, we could distinguish four major groups including the EPA-like genes, two VSHIT-containing groups, and another group with genes lacking EPA and VSHIT signatures. This grouping is less complex than proposed earlier using CBS138 data (seven clusters) (de Groot *et al.* 2008). The overlay between both grouping showed consistently that cluster I contained the EPA-like genes and that clusters V and VII and clusters II and III were grouped in two categories that include VSHIT-containing adhesins. The other clusters (IV and VI) are located in distinct branches, the latter being grouped in genes lacking EPA and VSHIT signatures. It is presently difficult to validate one or the other categorization, since no systematic functional analysis of all adhesins has been yet carried out with the exception of EPA-like genes (Zupancic *et al.* 2008). Now that a more extensive repertoire of adhesins is available from our studies, such analysis may be undertaken in the future.

## Supplementary Material

Supplemental material is available online at www.g3journal.org/lookup/suppl/doi:10.1534/g3.117.042887/-/DC1.
