# Supplementary material for: Comparative Genomics of Two Sequential Candida glabrata Clinical Isolates
Source: G3 (Bethesda). 2017 Jun 28;7(8):2413–26. doi: 10.1534/g3.117.042887 (PMC5555451; doi:10.1534/g3.117.042887)
Supplement: Supplementary file 12 [file 2413TableS1.docx]

**Table S1:** Primer used in this study

| Primer | Sequence |
| --- | --- |
| primer ChrD 691380 R | GTCCGGGAGCCATGAACAGTGTTACCGTCTGACTCAG |
| primer Chr D 690916 F | CGACGCCCGCTGATATGTGATCTCTTACTCGCCATCTG |
| primer Chr D 293039 R | GTCCGGGAGCCATGAGTTTTCATCCGGAGAGCTTAGT |
| primer ChrD 292312 F | CGACGCCCGCTGATACATCAAACGACGTAACTGTTGAA |
| primer Chr D 593851 F | CGACGCCCGCTGATAATACTCCCCTGCATGACGTATAT |
| primer Chr D 594348 R | GTCCGGGAGCCATGTCTTAGGAGGGGGGTAGTGATAT |
| primer Chr A 421584 R | GTCCGGGAGCCATGACAGAGAGAGAAGAGAGAGAGAG |
| primer Chr A 421080 F | CGACGCCCGCTGATATTTTTCACTTTTCGCTTGTCAAC |
| primer Chr C 2026 F | CGACGCCCGCTGATAAATCAAACTAATTCCCGTCACCA |
| primer Chr C 2302 R | GTCCGGGAGCCATGATGTTAGGAGCTGGAGTGATAGA |
| primer Chr C 5528 R | GTCCGGGAGCCATGCGATCTTTCCCTAAAACTGGTGT |
| primer Chr C 5154 F | CGACGCCCGCTGATACCATAATCACAAATGCCATGATC |
| primer Chr C 22727F | CGACGCCCGCTGATATTACCGTTAGAACCACCAGGATT |
| primer Chr C 23344R | GTCCGGGAGCCATGCACCATTGTCACCACAATCCCAT |
| primer Chr E 211285 F | CGACGCCCGCTGATAATAGTGTATGGCTCCGTGGATTA |
| primer ChrE 211599 R | GTCCGGGAGCCATGGATTAACCCTCTCATCCATTAGC |
| primer ChrE 290692 F | CGACGCCCGCTGATAGAGCTTTACCACAAACAAGCTTT |
| primer ChrE 291048 R | GTCCGGGAGCCATGGCACTTCCTGTTAATTGACTATT |
| primer ChrE 367114 F | CGACGCCCGCTGATAATCGATCTCCATGGAATTTGAAC |
| primer Chr E 367448 R | GTCCGGGAGCCATGAGAATTCCGTCTACACTCTTTCC |
| primer Chr E 464383 F | CGACGCCCGCTGATAAAGACTACTAACTCCCCTCCAGT |
| primer ChrE 464747 R | GTCCGGGAGCCATGTGACAAAAGTGGATAATCGAAGC |
| primer Chr E 574708 F | CGACGCCCGCTGATAACCTTCCACTCCTATCCCTTATC |
| primer Chr E 575105 R | GTCCGGGAGCCATGGTTCTGTGCTTTTGGTGGAATAT |
| primer Chr E 583338 F | CGACGCCCGCTGATATGTGGTCTCAACTTCCCTCTATT |
| primer ChrE 583715 R | GTCCGGGAGCCATGGCCTGGATATATGTATGGCCTTT |
| primer Chr E 695206 F | CGACGCCCGCTGATAAACCAATACTGTTATGATAGTCCA |
| primer ChrE 696521R | GTCCGGGAGCCATGAGACATCGTCTCCTTCTTCCCATC |
| primer ChrE 24555 F | CGACGCCCGCTGATAATAATGATGTCTCCATTAGAACCC |
| primer Chr E 31168R | GTCCGGGAGCCATGCTACCCGGGTATATATCGATGCTG |
| primer ChrF 91461 F | CGACGCCCGCTGATATGTATGATCAAACACCCGCAAAAA |
| primer Chr F 91899 R | GTCCGGGAGCCATGGCTCTTCTCATATATGGCACTCTC |
| primer ChrF 414202 F | CGACGCCCGCTGATAAACAGGAGCATGGAAAACAAAATG |
| primer Chr F 414410 R | GTCCGGGAGCCATGTTCATAAGCAGCGGAAGTCAAAAA |
| primer Chr F 465041 F | CGACGCCCGCTGATAAGGATCATCAGGAAGGGAAAATCT |
| primer Chr F 465579 R | GTCCGGGAGCCATGCTCTACATTGCTTTCTGTGAATCC |
| primer Chr F 488115 F | CGACGCCCGCTGATATCATTTCCATCTTACATCCGGGAA |
| primer Chr F 488335 R | GTCCGGGAGCCATGGAGAATCCTTTAGTAACCCGAACT |
| primer Chr F 513220 F | CGACGCCCGCTGATAGATAAAGTGAATGTTGCTCGGAAT |
| primer Chr F 513519 R | GTCCGGGAGCCATGGGCAAGTTCTTTAGATGGAATCAG |
| primer Chr F 751815 F | CGACGCCCGCTGATAATGAGTTCCCCCTTCACATCAATC |
| primer Chr F 752144 R | GTCCGGGAGCCATGGTAATCAATCCTTTTCCCTTCCAT |
| primer Chr F 919346 F | CGACGCCCGCTGATACCAAAAAACCACTGAGAGAGATAA |
| primer Chr F 919692 R | GTCCGGGAGCCATGGTATTGCTAGTAATCAGGGCATCC |
| primer Chr F 1159356 F | CGACGCCCGCTGATAATGTTCTAGCTCCCCGGGAATATT |
| primer Chr F 1159835 R | GTCCGGGAGCCATGTGGCGAAATTTGCTGTTAAGAGAA |
| primer Chr F 1551549 F | CGACGCCCGCTGATAAAAGGTGTCACTCCCTAACCATAA |
| primer Chr F 1551930 R | GTCCGGGAGCCATGAGCAAAATCCACTCACTTTACCAA |
| primer Chr H 97600 F | CGACGCCCGCTGATACTGGGGTTCTTCTGAGATCTAGTG |
| primer Chr H 197856 R | GTCCGGGAGCCATGCAAGTACCTAAAGAAGAGCATACG |
| primer Chr H 413360 F | CGACGCCCGCTGATATCTTCTGCTAATGACGCTGAACTA |
| primer Chr H 413656 R | GTCCGGGAGCCATGTAGGTAGAGCTTGCTGTTTCGTAT |
| primer Chr H 859099 F | CGACGCCCGCTGATATTGCATTTCTGACAACTCTCAAGG |
| primer Chr H 859539 R | GTCCGGGAGCCATGCAGGAACAGCTTGAAAGAGAAAAC |
| primer Chr I 277462 F | CGACGCCCGCTGATACAATTCCAAACAGAGCAAGGTAAA |
| primer Chr I 277912 R | GTCCGGGAGCCATGGCTGGGTCTTTGTTTCTTTTTCTA |
| primer Chr Jlong 932696 F | CGACGCCCGCTGATATGATCAAGAAACGTCAGAAGGTAT |
| primer Chr Jlong 933177 R | GTCCGGGAGCCATGAACTTCACTCATCTTACAGCTTCC |
| primer Chr Jlong 142416 F | CGACGCCCGCTGATACTTACCAAGTCCGTTCCTGAGCTT |
| primer Chr Jlong 142757 R | GTCCGGGAGCCATGCAGCGATTCATTTTTGCAACTTTT |
| primer Chr Jlong 532624 F | CGACGCCCGCTGATACATAACTGATAACGGCGTCTCTTA |
| primer Chr Jlong 532960 R | GTCCGGGAGCCATGCGTATTGGTGATATTGGCAATTGA |
| primer Chr Jlong 551646 F | CGACGCCCGCTGATAAAATTTATTCCCAGGCGATCTTCC |
| primer Chr Jlong 552035 R | GTCCGGGAGCCATGGATTTCCAGATTGTTCCTGCATAA |
| primer Chr Jlong 711000 F | CGACGCCCGCTGATACTTCACAAACATCTTCGTATCAGT |
| primer Chr Jlong 711355 R | GTCCGGGAGCCATGACAACAATCCACCAAAAGAAGAGA |
| primer Chr Jlong 1028665F | CGACGCCCGCTGATACTCCAACAATCCAAGTACCTTTTC |
| primer Chr Jlong 1029425R | GTCCGGGAGCCATGGAGAGACTGTATGTTCCACCAAAA |
| primer Chr Jshort 166331 F | CGACGCCCGCTGATATTCCTTTTCCTAACAGCATTTCCA |
| primer Chr Jshort 166542 R | GTCCGGGAGCCATGTTGTGGTGTAGTGATAGATGAGGA |
| primer Chr K 249251 F | CGACGCCCGCTGATAAGAGAGCAAGAGAGAGAGAGACTT |
| primer Chr K 249531 R | GTCCGGGAGCCATGATGCGTCGTCCTATTTATACTAGC |
| primer Chr K 465992 F | CGACGCCCGCTGATACGTCATTTACAAGCACTCGTTTTT |
| primer Chr K 466452 R | GTCCGGGAGCCATGTTCCCGGGATCTCTTGTTAGTTTT |
| primer Chr M 22681F | CGACGCCCGCTGATAAGCTCTGACAGTAGCTCCTACATC |
| primer Chr M 23414R | GTCCGGGAGCCATGTGCTCTGATTCACAGCTCAAGCGT |
| primer Chr M 110552 F | CGACGCCCGCTGATAGCAATATAACGCAAAGCACAATTT |
| primer Chr M 110989 R | GTCCGGGAGCCATGCATAACTACCCCTCCTCCTTTTTT |
| primer Chr M 338948 F | CGACGCCCGCTGATATCATATTACAAACAACCACCTCGT |
| primer Chr M 339342 R | GTCCGGGAGCCATGTTGAAGTGCTATCCTCCCCATATC |
| primer Chr M 855710 F | CGACGCCCGCTGATAACCGAGTATTCTTTTCGTCGTTTT |
| primer Chr M 856015 R | GTCCGGGAGCCATGGGCTTCATCAATTTTCCTACTCTG |
| primer Chr M 908616 F | CGACGCCCGCTGATATCCCTCTCTGACTATTGTGCTCTA |
| primer Chr M 909093 R | GTCCGGGAGCCATGGTGCAGCCTCTTTCTTATACTTTT |
| primer Chr M 993125 R | GTCCGGGAGCCATGTCTAGAGGAACACAACCGATACAT |
| primer Chr M 992723 F | CGACGCCCGCTGATAGACTCTGGATCTATGACACTGAAA |
| primer Chr M 1053402 F | CGACGCCCGCTGATAGGCAAACGTCGATTCTGAAATTAC |
| primer Chr M 1053736 R | GTCCGGGAGCCATGGTGAATGCTCCTTATATGTCACAA |
| primer Chr M 1182846 F | CGACGCCCGCTGATACCTGCGTAATATTCTGTGTACCTT |
| primer Chr M 1183228 R | GTCCGGGAGCCATGTGGATGTGCGGGTGTTTATATTAG |
| primer Chr M 1224321 F | CGACGCCCGCTGATATACAATATTTCCAGCCTGACTACA |
| primer Chr M 1224758 R | GTCCGGGAGCCATGTCACAATGCATACAGATCGAAAAT |
| primer Chr M 1260397 F | CGACGCCCGCTGATATTCCGTTACTTAGTTGGTGGAATG |
| primer Chr M 1260798 R | GTCCGGGAGCCATGGTGAGAATAAACCTGGCTAACATG |
| primer Chr M 1313882 F | CGACGCCCGCTGATAGCACTTAACTTTCTGTGATCTGTG |
| primer Chr M 1314318 R | GTCCGGGAGCCATGTTTCTTGGAAGTATGAGCCTGTTC |
| primer Chr M 1414916 F | CGACGCCCGCTGATATCCTCTCATTATGGGTACGTCTTT |
| primer Chr M 1415264 R | GTCCGGGAGCCATGATGTTTCGACGTCCATGAATTTCA |
| primer Chr M 156738 F | CGACGCCCGCTGATATGGAGTAGCAGTTGTAGAATACCA |
| primer Chr M 157089 R | GTCCGGGAGCCATGAGGACATCAGAATCGACTTATTGC |
| primer Chr C 178194 F | CGACGCCCGCTGATAACCCAACCTATGTATACAATGG |
| primer Chr C 178518 R | GTCCGGGAGCCATGGTCCAGATACGCAGTACTTCCT |
| primer Chr FLI 434689 F | CGACGCCCGCTGATATTCGGGCTCGGGATGTCCTCT |
| primer Chr FLI 435038 R | GTCCGGGAGCCATGTTGTATTGAATTTGAATTTGAA |
| primer Chr G 2171 F | CGACGCCCGCTGATACGAGATCCAGTCCGACTCAATG |
| primer Chr G 2597 R | GTCCGGGAGCCATGGCTACTGGACGACCACCAATGA |
| primer Chr G 227883 F | CGACGCCCGCTGATAAACATCAGATAAGACAAGACA |
| primer Chr G 228276 R | GTCCGGGAGCCATGGTTTGGCAATATCAGAAAATA |
| primer Chr G 736529 F | CGACGCCCGCTGATACGGTATGCTACAGCCTTGTGCC |
| primer Chr G 736950 R | GTCCGGGAGCCATGCCTAGTATGCTTGAAAGGTATG |
| primer Chr H 783172 R | GTCCGGGAGCCATGTAATGAACCCCAAAGTACTTAT |
| primer Chr H 783643 F | CGACGCCCGCTGATAGAAATGGGACTACGTAACAAG |
| primer Chr I 528673 F | CGACGCCCGCTGATACGTTACCGCCCTGAAACTCTG |
| primer Chr I 529081 R | GTCCGGGAGCCATGTGCGCCGTCAGCTTTGTCATC |
| primer Chr I 698274 F | CGACGCCCGCTGATACCATCTCCCTGAAGCACACGG |
| primer Chr I 698645 R | GTCCGGGAGCCATGGAGCAATACAAGTTTGCCTGG |
| primer Chr K 247216 F | CGACGCCCGCTGATAGCTGGTGCTGACCCAGAGATC |
| primer Chr K 247615 R | GTCCGGGAGCCATGGACATAACATCAAGTCCCAAA |
| primer Chr L 974295 F | CGACGCCCGCTGATACCCGATCCCAACCTCGATCAT |
| primer Chr L 974676 R | GTCCGGGAGCCATGGGAAAAGCACCTTTTAGGGT |
| primer Chr M 752022 F | CGACGCCCGCTGATACCCAGCCTTCCCACAGTCCC |
| primer Chr M 752387 R | GTCCGGGAGCCATGGGGCAGCCATCGCGTCGGGA |
| Primer Chr D 253027 F | CGACGCCCGCTGATATAGCATGGAACGTTTGGTGAA |
| Primer Chr D 253471 R | GTCCGGGAGCCATGCAGAAGTGGCCAAGGATGTAA |
| Primer Chr D 417786 F | CGACGCCCGCTGATAGCTTAGCATGATGTGTACTGTG |
| Primer Chr D 418188 R | GTCCGGGAGCCATGACGTGTCTTCTAAGGTTCCTTG |
| Primer Chr D 560615 F | CGACGCCCGCTGATATGAACCTCACACAATGGATGG |
| Primer Chr D 561035 R | GTCCGGGAGCCATGCTAAACACGGGAAATGGCTTC |
| primer ChrD 691380 R | GTCCGGGAGCCATGAACAGTGTTACCGTCTGACTCAG |
| primer Chr D 690242 F | CGACGCCCGCTGATACACTATCTCCACCATCACTGCTAG |
| primer Chr E 695098 F | CGACGCCCGCTGATAGTACATACATTATACATGTATAT |
| primer Chr E 696306 R | GTCCGGGAGCCATGGCACAGGCACCACCACCATCACC |
| Primer mito 14652 F | CGACGCCCGCTGATACCGCAAAATATATTGGAGCTGGAA |
| Primer mito 15382 R | GTCCGGGAGCCATGGACTTTCGTGTCCTTTATATATTACT |
| Primer mito 2315 F | CGACGCCCGCTGATACGTGAGGGAAAGTATGTAAATGG |
| Primer mito 9145 F | CGACGCCCGCTGATAGCTGGTACTGGTTGAACTGTA |
| Primer mito 2828 R | GTCCGGGAGCCATGATCGCACATAATCTGACTCCTT |
| Primer mito 9603 R | GTCCGGGAGCCATGAGTATAATTAATAGAACCAA |
| primer Chr D 681255 F | CGACGCCCGCTGATAATCTTACCTGGTTTGCAATTGAGTG |
| primer Chr D 681592 R | GTCCGGGAGCCATGGTGAAACGATTGAAAGGTCTTGTCT |
| primer Chr E 70697 R | GTCCGGGAGCCATGTTTCAATAGTAGCTGCTTGTACTGG |
| primer Chr E 70226 F | CGACGCCCGCTGATAATAAGACAGGAGTTTGCCCAGCTTT |
| LTR 1,167,371 F | TTGAATAGACATCGGAAGGTTGTT |
| LTR 1,167,985 R | AGCCCTAACTCGATTTCTTTTCTG |
| LTR 1,168,386-R | TTCCAGTAGCGGCTGGATTCAC |
| FLR1 608,069 F | GTCCGGGAGCCATGTGGCCTGGAGTATAAATTGACGAT |
| FLR1 608,555 R | CGACGCCCGCTGATATCAGTATTTGCAATTGTGAAGAGAG |
